# Implementation of simulation-based health systems science modules for resident physicians

**DOI:** 10.1186/s12909-022-03627-w

**Published:** 2022-07-30

**Authors:** Luming Li, Jessica M. Ray, Meghan Bathgate, William Kulp, Julia Cron, Stephen J. Huot, Ambrose H. Wong

**Affiliations:** 1 Department of Psychiatry at Yale School of Medicine, New Haven, CT 06519 USA; 2 The Harris Center for Mental Health and IDD, Houston, TX 77092 USA; 3 Department of Emergency Medicine, Yale University, 464 Congress Ave, New Haven, CT 06520 USA; 4 Educational Program Assessment at the Yale Poorvu Center for Teaching and Learning, New Haven, CT 06511 USA; 5 Department of Obstetrics, Gynecology and Reproductive Sciences, Yale University, New Haven, CT 06519 USA; 6 Department of Obstetrics and Gynecology, Weill Cornell Medical College, New York, NY 10021 USA; 7 Graduate Medical Education at the Yale School of Medicine, New Haven, CT 06510 USA

**Keywords:** Patient simulation, Graduate medical education, Health system science

## Abstract

**Background:**

Health system science (HSS) encompasses both core and cross-cutting domains that emphasize the complex interplay of care delivery, finances, teamwork, and clinical practice that impact care quality and safety in health care. Although HSS skills are required during residency training for physicians, current HSS didactics have less emphasis on hands-on practice and experiential learning. Medical simulation can allow for experiential participation and reflection in a controlled environment. Our goal was to develop and pilot three simulation scenarios as part of an educational module for resident physicians that incorporated core and cross-cutting HSS domains.

**Methods:**

Each scenario included a brief didactic, an interactive simulation in small-group breakout rooms, and a structured debriefing. The case scenario topics consisted of educational leadership, quality and safety, and implementation science. Learners from four residency programs (psychiatry, emergency medicine, orthopedics, ophthalmology) participated January – March 2021.

**Results:**

A total of 95 resident physicians received our curricular module, and nearly all (95%) participants who completed a post-session survey reported perceived learning gains. Emotional reactions to the session were positive especially regarding the interactive role-play format. Recommendations for improvement included participation from non-physician professions and tailoring of scenarios for specific disciplines/role. Knowledge transfer included use of multiple stakeholder perspectives and effective negotiation by considering power/social structures.

**Conclusions:**

The simulation-based scenarios can be feasibly applied for learner groups across different residency training programs. Simulations were conducted in a virtual learning environment, but future work can include in-person and actor-based simulations to further enhance emotional reactions and the reality of the case scenarios.

**Supplementary Information:**

The online version contains supplementary material available at 10.1186/s12909-022-03627-w.

## Background

The Accreditation Council for Graduate Medical Education (ACGME) has incorporated multiple competencies related to health systems science in residency training to highlight the importance of understanding complexities of systems in delivering effective and safe patient care [1]. Core competencies in practice-based improvement and system-based practice are important components of health systems science (HSS) [2].

Several reports have identified deficits among newly trained physicians in leadership skills for HSS [3]. One challenge to teaching HSS is that it encompasses multiple competencies in health care delivery, financing, communication skills, team-based care, population health, and the attainment of patient safety and quality, which can then be further divided to identify core and cross-cutting domains [4]. Within our institution, some ad-hoc didactic lectures and apprenticeship-type electives in healthcare administration are being conducted within several disciplines for resident physicians, but no site-wide formal training in HSS is currently in place. Experts have called for radical transformation and redesign of educational curricula that prepare trainees to lead executive teams and address challenges in rapidly evolving systems of care [2].

Curricula that have been developed in HSS within undergraduate medical education focuses on content knowledge and didactics, but less on experiential learning [5]. Although resident physicians are constantly placed in real-life situations that include many cross cutting HSS domains, they are rarely debriefed post-event or viewed through an HSS lens [6]. Simulation-based medical education is a tool that incorporates structured, skill-building learning experiences and has been used to teach physicians communication and non-technical skills. Simulation helps activate learners’ emotional or affective states, allowing for development of cognitive and communication skills necessary in clinical practice through an immersive and psychologically safe environment that ultimately leads to significant improvement in patient safety [7]. Studies have demonstrated feasibility in simulation to target healthcare organizational leadership and systems science for executives and managers [8]. Although case-based curricula for patient safety and quality improvement that involve discussions and simulation likely exist locally at various institutions, no standardized or widely used curricula for HSS that incorporates simulation currently exist for graduate medical education.

In this innovation report, we describe the implementation of a structured curricular module consisting of three simulation-based clinical case scenarios for resident physicians across four residency programs that integrate core and cross-cutting competencies in HSS using realistic situations that can occur in healthcare settings.

## Methods

### Theoretical background and curricular design

**Fig. 1:**
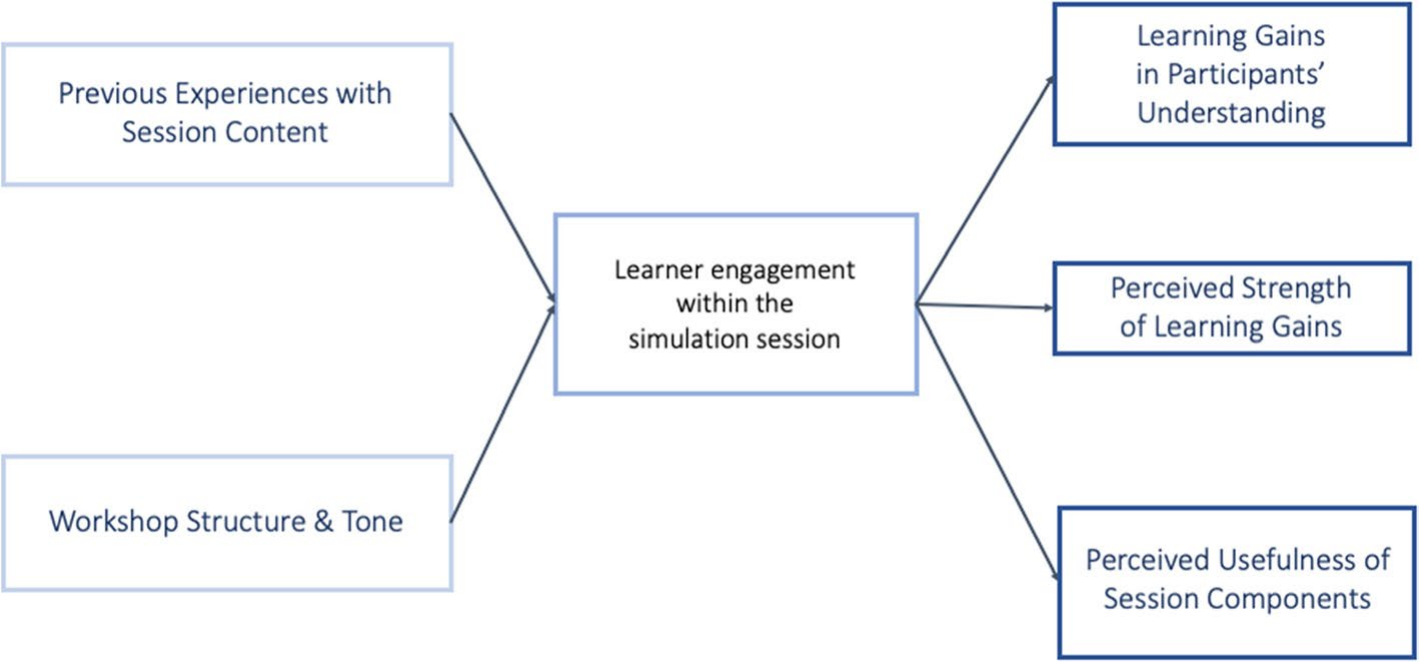
Overview model showing the factors contributing to learners’ session engagement and learning outcomes

To address the complexity of teaching HSS, we used an instructional and evaluation approach that was grounded and aligned to Self-Determination Theory (SDT) [9]. SDT emphasizes that learners' engagement and learning outcomes are directly related to their relationships with each other, perceived competence in a content area, and autonomy regarding their engagement. Our educational innovation reflects SDT by scaffolding participants’ knowledge throughout each curricular case scenario by incorporating a clear structure for respectful interactions amongst learner groups and allowing for a high degree of autonomy in how individuals enacted their role. SDT builds on the benefits of simulation-based learning by providing a more direct emotional connection to the learning materials and a setting for practicing interpersonal dynamics in a controlled environment. SDT also pairs with our evaluation approach, which explores participants’ perceived learning gains, specific changes in their understanding, and value of session components. Feedback provided by volunteer participants early in the design process was used to inform changes for subsequent workshop iterations (See Fig. 1).

Using this SDT-based approach, we embedded HSS core domains as well as cross-cutting domains to develop three interdisciplinary team-based case scenarios: 1) Implementation Science, 2) Education Leadership, and 3) Quality and Safety (see Additional file 1: Appendix 1). Each scenario detailed a clinical challenge with a specific systems-based clinical issue that participant teams are tasked to address. Residents had assigned roles within the scenario exercise that they portrayed and acted out during the simulation. We selected clinical contexts that would have applicability and familiarity across disciplines and training backgrounds. Scenario summaries and corresponding HSS domains are listed in Table 1. We initially included a fourth scenario focusing on health informatics and telemedicine but opted to drop it from the curriculum due to low level of interest from our graduate medical educational leadership.

**Table 1:** Case scenarios as related to core and cross-cutting domains

Case Scenario and Topic	1. Implementation Science	2. Educational Leadership	3. Quality and Safety
Scenario Summary	Implementing a depression screening care standardization tool as part of the Centers of Medicare and Medicaid Services (CMS) Merit-based Incentive Payment System (MIPS) at an outpatient clinic	Negotiation scenario to develop a new resident rotation and necessary hires to run a step-down unit, within the confines of a tight budget, staffing, and short timeline for implementation	Root cause analysis (RCA) of a serious safety event (SSE) concerning a high-risk fall patient who sustains a fall with injury when a nurse left to assist another patient
Participant Roles	Medical Director, Front Desk Staff Member, Clinic Provider, Informaticist, Patient/Family Representative, and Clinic/Nurse Manager	Vice Chair of Clinical Affairs, Program Director, Vice President of Clinical Operations, Chief Resident, and Medical Director of Hospitalist Service	Vice President of Patient Services, Clinical Program Manager, Subject Matter Expert, RCA analyst, Medical Director, and Quality and Safety Director
Main Teaching Points	Barriers to Implementation; Social determinants of health; Exploration, Preparation, Implementation, Sustainment (EPIS) framework [10]	Educational impact; Faculty development and resident supervision; “Best Alternative to a Negotiated Agreement” (BATNA) [11]	Systems-level factors; Quality improvement opportunities
Core Domains in Health Systems Science	Health care policy, financing, and management; Value-based care; Clinical Informatics	Healthcare structures and processes; Population and public health	Healthcare structures and processes; Health system improvement
Cross-cutting Domains	Evidence-based practice and Teamwork	Leadership and Change Management	Professionalism and Ethics

### Participant recruitment

Residency program directors at Yale School of Medicine were contacted by one of the authors (LL) requesting to have resident physicians participate as part of their core didactics. Directors of four residency programs representing procedural and non-procedural specialties (emergency medicine, adult psychiatry, ophthalmology, and orthopedic surgery) voiced interest given lack of formal training within their existing curricula and agreed to have their residents from across postgraduate years participate in the HSS curriculum as part of their programs’ core didactic series. Residents from all postgraduate years (PGY) 1–5 provided verbal consent for participation prior to the start of the simulation. This educational intervention was approved and deemed exempt by the Yale University Institutional Review Board. The institutional review board approved use of verbal consent for participation in the study given that the research presents no more than minimal risk of harm to subjects.

### Simulation sessions

We conducted the modules in a virtual learning environment using Zoom videoconferencing. Each session lasted two hours and included a ten-minute didactic that provided a conceptual framework of the main content topic. These ten-minute didactics incorporated the main teaching points highlighted in Table 1 and contained basic content knowledge that the participants could incorporate into the simulation activity. Participants were also provided anticipatory guidance on the simulation activity in a pre-briefing prior to the start of the simulation activity. This pre-briefing established expectations and for the learners including ground rules of engagement through role enactment within the exercise and the importance of psychological safety. After the pre-briefing, participants were divided into groups of four to six, in the form of a “breakout room,” where they participated in a 30-min tabletop simulation with designated roles. Each participant received a detailed description of the systems science problem at hand as well as the background and relevant goals of their assigned role within the simulation. When possible, a facilitator was present to answer questions and record observations of the activity to inform the larger group debriefing. Immediately following completion of the simulation, participants returned to a common room for a structured large group debriefing led by one of the study authors.

### Evaluation

Our evaluation approach reflected a developmental phase of applying HSS curricular content into graduate medical education, as no standardized approach has been well-established to evaluate the effectiveness of HSS curricula. At the start of each session, participants were asked about their prior experience with the session content via an electronic survey to identify the perceived needs by the specific learner group undergoing the simulation-based case scenario. At the end of each session, participants were asked to complete an anonymous post-intervention survey-based evaluation. The evaluation incorporated a core set of Likert-scale and qualitative questions that was adapted to apply to all three case scenarios (See Additional file 2: Appendix 2). We calculated means and standard deviations for responses to each survey question, percentage positive responses (inclusive of responses with 3, 4 and 5 on the Likert scale). Narrative free-text survey responses were collected and coded using directed content analysis techniques [12] to derive recurrent themes.

### Curricular and evaluation iteration

In building the three simulation modules, several iterations occurred to the curricula, debriefing approach, and evaluation early on the design phase. These included limiting the total number of roles and group sizes incorporated into simulation, as higher numbers of roles made it difficult to administratively manage the small group breakout room simulations. In addition, volunteer participants who helped with piloting and testing of the modules provided early feedback that they received the most benefit from the simulation and debriefing portions, so the didactic portion was shortened to highlight the key content information needed to participate in the simulation. Surveys were developed using an iterative refinement process led by a psychometric expert (co-author MB) and tested on a group of volunteer resident learners prior to formal launch.

## Results

A total of 95 resident physicians across postgraduate years (PGY 1–5) representing > 95% of all emergency medicine (*n* = 41), psychiatry (*n* = 24), ophthalmology (*n* = 12), and orthopedic surgery (*n* = 18) trainees at our institution participated in our curricular modules between January to March 2021. We conducted each of the three scenarios with residents from two different specialties. In the needs assessment survey responses (*n* = 95), the majority of participants responded “not at all” to “moderate” when asked about familiarity with the session content (72%), prior training (93%), and experience implementing structural changes (94%). This pattern indicated a clear need and room for growth among participants on the HSS topics covered in all three case scenarios.

Table 2 describes demographic data of our post-session survey respondents (*n* = 66, 69.5% response rate). Table 3 summarizes results from our post-session survey, which consisted of mostly positive feedback. There were not major differences in responses between participants from different specialties that attended the same scenarios. Coded qualitative responses resulted in three overarching themes (see Table 4). Emotions and reactions to the session (Theme 1) were positive overall, especially with regards to the interactive role-play format allowing for immersion within a health system science context. Recommendations for improving feasibility and applicability of the module (Theme 2) included participation from non-physician professions, distillation of core concepts with a post-session handout, tailoring of scenarios for specific disciplines/roles, and more directed support from facilitators during the breakout. Participants also described specific instances of knowledge application and transfer to the bedside (Theme 3) via incorporation of multiple stakeholder perspectives, effective negotiation by considering power/social structures, and enactment of system change using incentivization that considers downstream impacts on frontline staff.

**Table 2:** Post-session survey respondent characteristics

	**Overall**	**Implementation Science**	**Educational Leadership**	**Quality & Safety**
**Residency Specialties**		Psychiatry, Emergency Medicine	Psychiatry, Ophthalmology	Psychiatry, Orthopedic Surgery
**Sex**				
N	66	34	16	16
Male	36	22	6	8
Female	20	7	7	6
Blank/Prefer not to say	10	5	3	2
**Race/Ethnicity**				
N	66	34	16	16
White	34	24	7	3
Asian	13	7	4	2
Black	5	2	1	2
Latinx/Hispanic	2	1	1	0
Blank/Prefer not to say	12	6	2	4

**Table 3:** Post-session survey outcomes

Scale and Measure	Overall	Implementation Science	Educational Leadership	Quality & Safety
**Perceived Learning Gains**				
N	66	34	16	16
Mean (SD)	3.89 (0.77)	3.68 (0.77)	4.19 (0.54)	4.06 (0.85)
% positive	95%	94%	100%	94%
**Usefulness: Didactic**				
N	66	34	16	16
Mean (SD)	2.85 (0.87)	2.72 (0.98)	3 (0.89)	2.94 (0.57)
% positive	71%	64%	94%	81%
**Usefulness: Breakout Room (Simulation)**				
N	66	34	16	16
Mean (SD)	3.86 (0.94)	3.79 (1.04)	3.88 (0.81)	4 (0.89)
% positive	92%	91%	81%	94%
**Usefulness: Group Debriefing**				
N	66	34	16	16
Mean (SD)	3.5 (0.9)	3.5 (0.99)	3.69 (0.95)	3.44 (0.63)
% positive	88%	88%	81%	94%

% positive refers percentage of those who rated 3, 4, 5 on a 5-point Likert scale (1: No, not at all; 2: No, not really; 3: Moderately; 4: Yes, somewhat; 5: Yes, absolutely)

**Table 4:** Directed content analysis of narrative text responses in post-session survey

Qualitative Themes	Domains & Concepts	Sample feedback quotes
1. Emotions & Reactions to Session	Overall satisfaction and enjoyment of simulation experience despite feeling challenged due to unfamiliar material	“I think it was excellent, small groups are great because it allows for active participation, even if we all felt stretched having to work through something we haven’t done before.” “Breakout rooms were interesting and different, appreciated how it was structured and really enjoyed it.”
	Role playing is uncomfortable and demanding but provides participants the opportunity to situate their learning within health system contexts	“We fell into the roles as time went by, and it felt a lot more natural after that, but it did take a few minutes, I feel, to get into it. It made me realize I don’t envy medical directors. I would not want that role in real life, having to please everyone but also respect the bottom line.” “It was really awkward. I don’t think I could have had that conversation as a real person. It was nice to have my fake character to hide behind, to have a first try at doing this stuff before having to do it in real life.”
	Virtual format can be awkward due to need for turn-taking and limitations in interactivity	“I felt like the Zoom format made it a bit awkward and we had a lot of silences because it impeded free-flowing conversations and really getting into our roles.” “The small group session was a bit tough on Zoom format, I wonder if we can do this in person things would be smoother.”
2. Feasibility/Applicability of Session and Recommendations for Improvement	Representation and participation from other professions/disciplines would improve fidelity/experience	“More representatives from actual nursing staff…would make the priorities/pitfalls from each stakeholder more realistic.” “Use actual mix of professions. I felt like I was pretending to be a nurse and didn’t really know what they would feel or be worried about.”
	Distilling core concepts/teaching points for participants would help translation and long-term absorption of knowledge	“Give us simple handout boiling down takeaway concepts. I’d like to refer to them again in the future.” “Provide more examples of practices to change implementation and some of the case materials by email."
	Tailoring of case content/environment to the specific discipline/role of trainees would make simulations more realistic	“Make it more in terms for the emergency department…overall the activity helped show challenges for the outpatient setting but less for the hospital.” “I would like to think more about what my role would be as a surgeon and how I would respond to a serious safety event in the operating room.”
	Provide direct support/interaction with session facilitators would help the breakout simulation experience	“Would have been helpful to have a knowledgeable facilitator during the breakout with us…we were having trouble with the budget portion of the scenario.” “Have facilitators in each group please so they can answer questions as we go since the timing was so tight.”
3. Transfer of Knowledge to Bedside	Incorporating multiple stakeholder perspectives is complex and involves negotiation of competing priorities	“Made me think more about my current working environment…helped me understand how challenging it is to work with a multidisciplinary team and still meet timelines, especially thinking about IT support and the budget needed to make it happen.”
	Successful negotiation requires attending to social/power dynamics and use of practiced techniques like "Best Alternative to a Negotiated Agreement" (BATNA)	“Have to be considerate of others’ priorities…I work on a unit with doctors, clinical psychologists who are PhDs, APRNs, and counselors, each with their own stakes and in some cases unions.” “I feel like I have a good idea of how I would organize and carry out one of these processes using these techniques now, especially with administrators and non-MDs.”
	System change is affected by barriers at multiple levels of care delivery and can be influenced by type/nature of incentives	“Interesting to think about the practice of negative and positive incentives.” “Consider how to improve staff buy-in with motivating practice and making sure new practices in “workflow” don’t add to work burden for our nurses.”

## Implications for practice

In this innovation report, we developed simulation-based case scenarios to teach core and cross-cutting domains in HSS that were feasibly applied to residents in both medical and surgical subspecialties. We found that participants reported limited knowledge and familiarity with HSS topics, despite ACGME requirements. This may be due to the fact that (1) the content is not currently being taught in a structured way, and (2) HSS skills require an experiential method like simulation to effectively grasp, similar to other non-technical skills (e.g., end-of-life discussion, leadership and communication) [13]. We also found that participants reported that they most preferred the simulation-based portion of the module. Qualitative feedback from learners remarked on the challenges and gratification of developing rapid team cohesion to solve a health system challenge within the breakout format, mimicking the skills needed to effectively coordinate disparate stakeholders in temporary or ad-hoc groups [14]. Our pilot implementation was supported by a small foundation grant which allowed for a part-time research associate to administer and organize the sessions and some dedicated effort by a simulation specialist to lead the debriefings. Attempts to pilot similar curricula at other organizations may benefit from dedicated finances to similarly provide administrative support and simulation expertise for optimal outcomes.

Important next steps for the work include expansion and testing of the modules to additional specialties. In addition, the simulation module format may serve as a template for additional modules to be developed covering more content areas in HSS. Further piloting of the entire set of modules in each specialty residency cohort will be able to assess specialty-specific needs and content applicability. Our sessions incorporated trainees across multiple years of training level within the same session, which did not appear to hinder learning. In fact, some residents remarked positively on how the assignment of roles within the breakout often subverted the usual professional hierarchy amongst participants (i.e., a more junior resident would act as medical director while a chief resident would act as a nurse) which added humor and novelty to the experience.

The simulation modules were created using a virtual learning environment due to social distancing requirements at time of implementation and attempts at accommodating disparate trainee schedules, but participants remarked on some challenges to engaging with each other during the breakout session via the Zoom videoconferencing format. A future direction may be to use in-person tabletop sessions and actors to augment psychological realism of interpersonal interactions that occur during simulation. Additional approaches to evaluation, including incorporating assessments such as a situational judgment test and additional surveys to assess long-term impact post-residency can be developed and used to more carefully assess learner knowledge and application into clinical practice. In our next step of implementation, we aim to integrate our module within an institution-wide chief resident forum to target learners who are most likely to have jobs that include HSS in their day-to-day work after graduation and pilot our scenarios in other nearby institutions.

## Conclusions

In conclusion, we found that interactive, simulation-based learning modules in HSS were feasible to develop and apply to diverse physician trainee cohorts. Our evaluation showed that residents expressed lack of baseline content knowledge on multiple topics in health systems science, and that learning about core and cross-cutting domains using simulation-based modules produced perceived learning gains related to systems-based practice and care quality and was valued by participants.

## Supplementary Information

**Additional file 1:** **Appendix 1. **Case Scenarios.

**Additional file 2:** **Appendix 2. **Post-survey template items

## Data Availability

The datasets used and/or analyzed during the current study are available from the corresponding author on reasonable request.
