# Supplementary material for: Implementation of simulation-based health systems science modules for resident physicians
Source: BMC Med Educ. 2022 Jul 30;22:584. doi: 10.1186/s12909-022-03627-w (PMC9338604; doi:10.1186/s12909-022-03627-w)
Supplement: Supplementary file 1 — Additional file 1: Appendix 1. Case Scenarios. [file 12909_2022_3627_MOESM1_ESM.docx]

**Appendix 1. Case Scenarios**

**Module 1: Implementation Science**

Objectives:

1. Recognize the complexity in implementing a care standardization challenge
2. Analyze barriers and facilitators in implementing a care standardization change
3. Identify the multiple stakeholders who need to be engaged for the change to occur
4. Assess organizational readiness of change
5. Characterize the steps and methods to engage/negotiate with multiple stakeholders
6. Employ change management techniques to implement the care standardization change in the module

Target Audience: Residents across different Medical Subspecialities

Case: *Implementation Science Challenge*

Instructions for Part I:

For Part I, you will use a concept map in order to have a comprehensive understanding of the different factors that influence this module.

**Pre-Assessment: You will be given a Qualtrics link to review Part I of the case and answer basic questions related to the case.**

**PART I**

You are a medical director of an outpatient clinic in a small city in Northeastern U.S. As part of your clinic responsibilities, you’re tasked with implementing new clinical metrics as part of national patient safety/quality programs. A measure that has come across your desk for “Depression Screening,” that was announced as part of the Centers for Medicare and Medicaid Services (CMS)’s Merit-based Incentive Payment System (MIPS) Program. Starting in 6 months, the clinic would be expected to utilize an evidence-based depression screening tool and ensure that all patients cared for at the facility would have a standardized depression screening in place. The measure also specified the interval for screening to be annual.

The clinic is a primary care clinic that includes 30 clinical providers, including 10 physicians (many are part-time), 9 APRN/PAs, 5 nurses, 4 medical technicians, a social worker, and a part-time psychiatrist. The clinic currently has approximately 23,000 unique patients as part of the practice and is affiliated with an academic medical center. As such, there are at times rotating residents who see outpatients as part of their clinical training. The clinic is part of an academic medical practice, where a number of the physicians teach and have faculty appointments. The clinic is not considered a federally qualified health center and is historically under-resourced in terms of the number of social workers and psychiatrists supporting the clinic for patients with mental health need. The clinic is situated in a diverse city, including a number of older patients and patients with multiple medical conditions.

The clinic utilizes the academic medical center’s electronic medical record, which has informatics capabilities, although no in-built depression screening tool at the moment.

**Answer the following questions:**

1. What are some potential barriers to implementation?
2. Who would be impacted by this implementation?

Instructions for Part II:

For Part II, you will break up into small groups to delve deeper into the implementation of the depression screening tool. Your small group will each be given a specific role as related to the implementation, with instructions and details on the role. The group is tasked to meet together in order to work on the implementation plan for rolling out the evidence-based depression screening tool. As a clinic, the team has decided on using the Patient Health Questionnaire (PHQ)-9. After labor intensive chart auditing, the clinical team was able to obtain baseline data on a sample within the large primary care practice. Chart auditing revealed that only 30% of individuals had some depression screening during their annual visit, and of that, only 15% utilized a depression clinical scale for a standardized depression screening. The findings also suggest that Black patients had a 50% lower number of depression screenings than comparable sample of white patients.

**PART II**

**Small group activity: You will be assigned a unique role as part of a group within a Zoom Breakout Room.**

Medical Director of Clinic:

- You manage the clinical operations for the clinic and are in charge getting the different clinical providers bought-in to the new screening tool. You know that a number of your providers struggle with using the electronic medical record efficiently and will likely resist the implementation of the screening tool. As the depression metric is being launched, you are struggling to get resources to the metrics needed to successfully track baseline use of a standardized depression screen. Historically, the organization has mostly used pen/paper to do standardized scales, scanned into the chart. You helped complete the chart auditing as part of baseline data gathering, but do not believe that labor intensive chart auditing will be a sustainable way to gather data in the future.

In talking with colleagues outside your institution, you know that there are already built tools available for depression screening. You are frustrated that there is not a built-in PHQ-9 scale, and the organizational informaticist being utilized for this project is historically very slow at tackling electronic medical record build projects. You’re discouraged by the clinical care variation found during the chart audits and hope that a standardized process for depression screening will reduce the care gap and differences in care based on racial demographics. As a result, you are interested in advocating for data management resources and a clear path for measuring success in the depression screening project.

Clinic Front Desk Staff Member:

- You are a front desk staff member who helps schedule patients for visits and have historically helped patients complete a depression screening scale prior to their visit. This scale has been scanned into the chart, although sometimes the lag can cause frustration by clinical providers who are seeing the patient that day. You wish that there was a way for the patient to fill out the depression screening information prior to the patient coming into the clinic. However, up until now, you have not been able to convince clinic leadership to send the PHQ-9 to the patient as part of the pre-arrival intake packet. Now that there’s a new clinic manager, you’re more hopeful that the screen might be available to send prior to patients arriving to the clinic.

Clinical Provider (MD/APRN)

- You are a direct care provider, and care for a panel of approximately 1500 patients and have been feeling stressed by the added paperwork needed for “clinical quality requirements.” You just heard from the clinic medical director that a new depression screening needs to be completed on all patients and find it dubious how this will happen without added physician burden of typing in all the PHQ-9 scores in the note. Although you know that depression screening is important, you don’t want to be the one needing to administer the clinical scales. You already feel like there is a big burden on physicians to document on many aspects of patient wellness and related metrics, and believe that a standardized note template with PHQ-9 questions pre-populated in the chart would be helpful to reduce work burden.

In addition, although the clinic has a part-time psychiatrist, you’re worried that the new policy will lead to a large number of patients screening positive and needing specialized treatment. You’ve found it hard given the clinical resources to get patients mental health treatment. As a result, you’ve been filling in as the primary care provider, and giving basic antidepressant treatment although you don’t feel that this is adequate. You have had too many experiences where mental health treatment has been inadequately screened and treated in the primary care clinic, given the high number of other tasks by primary care providers.

Informaticist:

- You are the electronic medical record informaticist and help support builds for clinical projects involving new updates. You have been doing your job for 2 months, and there’s been high turnover in your department. Although you wish to fulfill requests quickly, your skills and abilities make it difficult, as you have not been sent for specialized training. At the moment, you are stuck navigating a number of new builds, including the depression screening, as well as multiple other requests. Two staff members in your department just quit, and you know you would need more help in order to successfully complete the project build.

Patient/Family Representative:

- You are part of a committee that represents patients and families in various clinic projects and continue to receive care in several clinic locations within the academic medical center group practice. You were asked by the medical director of the primary care clinic to help with design the implementation of the depression screening question. As a patient, you’ve been asked to do multiple scales and clinical surveys prior to visits and hope to provide feedback on designing a process that doesn’t require too many questions unless necessary. For depression screening, you’ve had family members complain to you that they would fill out the survey, but the provider does not review it, or asks the questions again during the visit because the survey wasn’t scanned in time for the provider to review the results. In addition, you’ve had other friends in the community who have told you that depression screening wasn’t even done, and they’ve had visits where depression was not even talked about in their annual visit.

Clinic/Nurse Manager:

- You are in charge of the staff of the clinic, outside of the medical providers. This includes the medical technicians, nurses, clinic front desk staff, and social worker. You were just promoted to this clinic manager role approximately 1 month ago, and previously worked at a different clinic before moving to the primary care clinic. You are still developing relationships with the clinic staff and have wanted to make clinical changes that help with efficiency and clinical care delivery. Of what you know so far, you realize that the clinic staff are frustrated with clinical workflows in place and have not had significant experiences redesigning workflows. You hope to be able to provide guidance to the teams and help with redesign workflows to help with some of the inefficiencies. You just became aware of the clinical depression screening policy and believe that this would be an important project for the team to complete as a team-building opportunity.

One of your biggest worries is that the primary care clinic has annual sunk costs, given the high numbers of Medicare and Medicaid patients in the patient mix. You know the broader clinic administration would be willing to invest some resources to help the primary care clinic meet its regulatory demands and drive value-based payment earnings, although you’re concerned about the practice’s long-term sustainability.

**Answer the following questions:**

1. What is needed to implement the depression screening tool?
   1. What are the necessary steps for implementation?
   2. How does your team plan to carry out the necessary steps?

**Module 2: Educational Leadership**

Objectives:

1. Understand the role of Best Alternative to a Negotiated Agreement (BATNA) in negotiations
2. Identify how individual stakeholders are impacted differently by decision-making
3. Using a simulation case, experientially learn about arriving at key decisions in a multi-entity negotiation case

Target Audience: Residents across different Medical Subspecialties

Case: *Education Leadership Challenge*

1. **Education Leadership Challenge:** A residency program director was requested by hospital administration to help staff a newly proposed step-down unit (SDU). The residency program director (PD) was tasked with creating a new rotation which will staff this new clinical service. In doing so, the PD must balance the educational needs of trainees with the clinical needs of the hospital.
   1. Individual roles include Vice Chair of Clinical Affairs, PD, Vice President of Clinical Operations, Chief Resident, and Medical Director of Hospitalist Service
   2. Team will need to decide on whether or not to move forward with a SDU rotation, through negotiations and discussion as relevant to training opportunities, staffing/operations, and financial costs.
   3. Team will be provided with individual roles, and tasked to navigate political/interpersonal conflict challenges that affect resident education and the establishment of a new resident rotation

Instructions for Part I:

For Part I, please read the following case and respond to the questions below.

**PART I**

You are the residency program director for internal medicine. The vice president of clinical operations recently talked to you about converting the new wing of the hospital into a step-down unit.

Due to recent increases in volume of intensive care patients, the need for a step-down unit has become a more pressing concern for hospital leadership. The new wing was selected to be located near the intensive care unit so that patients can be transitioned smoothly from one setting to the next. Although construction for the new wing has already started, the staffing plan for the unit is still being developed.

You were asked to assist with staffing of the step-down unit by creating a new resident rotation in the unit. The hospital has historically lacked a step-down unit, and you believe that a new clinical rotation in the step-down unit would likely provide a meaningful educational opportunity for the residents. In order to fully staff the unit with resident and teaching faculty, 4 residents would need to be on service (two daytime with one PGY2/3 and two interns, and one PGY2/3 overnight), at any given time. Residents in the program already have rotations in place and would need to give up rotating on a different service in order to staff the step-down unit as proposed.

However, the vice president only approached you this week, and you really wish you had been involved in conversations earlier. Due to being involved later in the process, you have not had a chance to tell any of the residents nor connect with the faculty members in the internal medicine department about the need for a new staffing plan for the step-down unit.

You first seek to identify the stakeholders who would be impacted by a new resident rotation in the step-down unit.

**Answer the following questions:**

1. Which stakeholders should be involved in creating a new resident rotation?
2. How would the new resident rotation impact existing rotations and operations?

**PART II**

Instructions for Part II:

For Part II, you will break up into small groups to delve deeper into the educational leadership challenge of creating a new step-down rotation. Your small group will each be given a specific role as related to the task of developing a new resident rotation, with instructions and details on the role. The group is tasked to meet together in order to negotiate on the feasibility of a new resident rotation and staffing plans for the new step-down unit. The team is asked to develop a balanced plan weighing the benefits and potential concerns for creating the new rotation, as well as mechanics and staffing related to the rotation creation.

**The team is tasked to identify and decide on the following:**

- **Educational Changes:**
  - No Change (No New Rotation)
  - Add to Total Number of Residents in the Program
  - Substitute Elective Time
  - Substitute Time from Another Inpatient Rotation
- **Staffing for the SDU:**
  - Hospitalists
  - Residents/Teaching Faculty
- **Start Time for Changes:**
  - 8-16 weeks
  - New academic year (16+ weeks)

**Proposed Staffing Options (based on a $0.75M budget):**

| **Daytime** | **Nighttime** |
| --- | --- |
| 3 residents + 1 teaching faculty | 1 resident overnight with cross-covering teaching faculty (teaching faculty not paid extra for cross-coverage) |
| 2 hospitalists* | 1 hospitalist overnight |

*Note: APPs have not historically practiced at the institution; hospitalists cannot also be teaching faculty, as the teaching faculty and hospitalists have different pay and reporting structures.

- **Budget for Project^+^:**
  - $0.75M
  - $1M
  - $1.25M

^+^Note: Staffing costs: $125K (resident, salary + fringe); $250K (hospitalist or teaching faculty, salary + fringe)

**Residency Program Director:** You are the program director for internal medicine. You would like to see the proposed step-down unit host a teaching team that includes both residents and teaching faculty. However, you are worried that the existing residents will not be thrilled that a new rotation is created, especially since you have not told them about the rotation. You’ve recently updated your chief resident group about the goals and project, and the chief resident is cautiously optimistic that residents would be interested. You will be bringing one of the chief residents to help provide a resident perspective during the meeting. You hope to start the new rotation in the following academic year (4 months away), so you have time to prepare for the implementation of the rotation and to work with your chief residents to coordinate the staffing and scheduling. With regard to staffing, you would not like to hire new residents, as expanding the size of the residency program would require consideration on the quality of education as well as available rotations outside of the step-down unit rotation. In addition, you would have to consider obtaining ACGME approval if additional residents were added to the program. As it is, the program already has >80 residents for a 3-year program. Thus, you would like to potentially incorporate the new SDU rotation alongside existing inpatient rotations or elective time, but would need the hospitalists to expand coverage to another medical unit in order to meet the staffing needs if residents were removed to staff the SDU. In addition, you believe the SDU rotation may be helpful in recruiting future residents, as not having a SDU has been an educational gap for trainees.

Although not directly related to your role, you would be supportive of hiring additional hospitalists to make the new SDU rotation work. You are also supportive of having additional teaching faculty be hired, although you recognize additional teaching faculty may not be absolutely necessary (since some teaching faculty would be relieved of current inpatient medical unit duties if new hospitalists are hired).

**Chief Resident (Optional):** You are acutely aware that the SDU rotation would provide helpful continuum of care from the ICU level to the inpatient medicine units. You believe that residents would appreciate the ability to rotate in the SDU, as long as caseloads are reasonable and the team structures are adequately staffed. In addition, you know that two of the medical units (renal and liver services) are teaching services that have had multiple complaints from residents about inadequate faculty support, which has generated concern among the chief resident group. Residents are concerned about volume of patients and not receiving enough teaching time, as well as not having faculty with expertise in medical subspecialty medical floors. You believe that the proposed team structures (3 daytime residents + 1 teaching faculty, and 1 nighttime resident with cross-covering attending) would allow for teaching time and appropriate case load. However, you are worried about making sure that other program chiefs for the internal medicine prelims and interns will be on-board with a new SDU rotation.

From your vantage point, you are supportive of the new SDU rotation being created, and would want to advocate for time to be substituted from another inpatient rotation, instead of elective time, since the resident group would want to have adequate time throughout residency to explore fellowship options. You have no interest in budget concerns, but would support use of funds to hire additional teaching faculty, especially in areas that have weak teaching faculty coverage on the medical floors.

**Vice Chair of Clinical Affairs (Teaching Faculty Lead):** You help lead the teaching faculty group in your role as Vice Chair of Clinical Affairs for the Internal Medicine Department. Currently, you are experiencing a very tight budget cycle, with difficulty getting hospital support for new academic medical positions for teaching faculty. You are excited about the new SDU opportunity, after having heard from the vice president of clinical operations that there is interest by the hospital to start a new teaching service. Historically, the hospital leadership has not openly supported academic teaching roles partially because of needing to support some non-clinical time for teaching faculty. You believe the SDU proposal may be an opportunity to ask for 2-3 new faculty roles from the hospital administration.

Although you have not talked to the internal medicine program director, you would wholeheartedly support the expansion of the teaching service to include the proposed SDU. Although you recognize that teaching faculty may not be as interested in SDU service due to acuity, you believe you can convince or hire faculty with interests across the intensive care continuum with the right funding and timing. There are currently 1-2 residents graduating from the institution with interest in becoming an academic teaching faculty that you would like to hire, and are still working out the funding packages for. They are also looking at other opportunities, so having a funding line to support their hiring would really be a win-win for all. Hires beyond the 1-2 graduating residents would likely be hard within a short time period, and you would likely need to wait for another academic year and application cycle in order to fully recruit. You don’t have strong opinions about how the residency program works out the SDU rotation, and would be supportive of ideas that come from the residency program director. Unfortunately, this year, you don’t have strong other options for obtaining funding to hire the additional teaching faculty.

**Medical Director of Hospitalist Service:** You help lead the hospitalist service, and have been interested in expanding your staff since many of your staff are overworked. You are currently hiring for two open positions, and are in the final stages of giving offers. You have a large staff with some recent hires as well as seasoned hospitalists, many of whom trained at the institution.

With the new proposed clinical opportunity of the SDU, you are both eager to find out whether the budget can be used to help expand the hospitalist service. However, you’re concerned that the SDU may not be as popular of a service for the hospitalists because of the acuity, although you would be willing to balance the clinical load with lower census and adequate staffing. You had heard from the vice president of clinical operations that there may be interest in the teaching service helping staff the SDU. You would be supportive of a teaching service covering the SDU, and would be agreeable to placing hospitalist staff on other medical units to offset the teaching staff needed to create a SDU teaching service. However, you would want to hire for at least 4 new hospitalists (3 hospitalists, and 1 back-up hospitalist, equaling $1M) in order to cover another medical unit or the SDU. You are reluctant to hire <4 hospitalists as you have needed to scramble to find coverage for vacations and sick call-outs when staffing was exactly per budget proposals. This has been a challenging problem for you during the last year, during which the hospitalist service has been short-staffed. After the two new hires, you will then have a fully staffed service. Although you may be able to provide temporary coverage for the new SDU or another medical unit through a per-diem and moonlighting pool, such an arrangement would likely need to be temporary as coverage would require at least 3 new hospitalists.

Although you are open to having additional teaching staff be hired, you would not want to do so at the expense of hiring hospitalists. You are willing to have the hospitalists directly provide service to the SDU if a teaching service rotation is not easily agreed upon.

**Vice President of Clinical Operations:** You are tasked with implementing the SDU, and believe that a teaching service would be ideal, as patients are more acute than normal hospital-based units, and a teaching service may provide better quality of care since trainees are involved in care plans. However, you would like to do implement staffing at the lowest cost possible, ideally within a $0.75M budget for increased medical staffing. You know that the medical director of the hospitalist service and the vice chair of clinical affairs will both be interested in new positions within their own service areas. Although you are able to pay more than $0.75M, the most you can spend on the project is $1.25M. Hospital finance has requested that you try to make the budget work as close to $1M as possible, since there would be many additional staffing costs associated with the new SDU, beyond medical staff costs.

You are aware the hospitalist and teaching faculty are non-overlapping services/personnel with different reporting structures. Your preference is to hire more hospitalists, as you know there’s been challenges with medical staff there feeling overworked. In addition, the teaching faculty need compensation for non-clinical aspects of their role, including protected academic time. However, you do recognize that starting a teaching service will likely require additional teaching faculty, and therefore, are open to having a mix of teaching faculty and hospitalists hired. Ideally, you’d like to have the unit fully staffed within the next 8-16 weeks. However, you may be able to temporarily work with the medical director of the hospitalist service to help provide temporary staffing until the new academic year if a new SDU rotation is to be implemented.

If the teaching service is too difficult or expensive to implement, your best alternative is to work directly with the hospitalists to provide service to the SDU and the start time of the project would be targeted for 8-16 weeks.

**Answer the following questions:**

Please work with your team on deciding on the following issues:

- Educational Changes
- Staffing for the SDU
- Start Time for Changes
- Budget for Project

Use the worksheet titled “**Decision Sheet for SDU Rotation Negotiations**” provided to indicate decisions made. The meeting among the different stakeholders does not need to be structured, but the team should work out the decisions through a vote or joint decision before the end of the time allotted. Majority is >4 of the stakeholders.

**Module 3: Quality and Safety**

Objectives:

1. Identify steps to respond to a serious safety event
2. Define stakeholders who are important to include in a root cause analysis
3. Detail key events that took place (both the actual events and ideal events)
4. Decide on specific interventions to prevent future serious safety events in the same category

Target Audience: Senior Residents across different Medical Subspecialities

Case: *Quality and Safety Challenge*

Instructions for Part I:

**PART I**

You are the safety coach for a mid-sized community hospital. Your team shares with you that a safety event occurred, and used the Situation, Background, Assessment, and Recommendation (SBAR) template to share about the specific case.

**Situation:** Patient fall with injury.

**Background:** 72-year old female with multiple medical and psychiatric comorbidities presented to the ED from an extended care facility with a week of poor oral intake, as well as nausea/vomiting. Patient was admitted to the medical ICU with a diagnosis of metabolic/respiratory alkalosis with anion gap metabolic acidosis. Once stabilized, the patient transferred to another medical floor unit, with an optimistic discharge date of 24-48 hours dependent on COVID-19 negative testing. However, on the next day, a nurse had started assisting the patient to the bathroom but briefly left to go “respond to another patient yelling" and heard the patient fall. The patient subsequently reported right-sided hip pain. An x-ray revealed a right-sided hip fracture. The patient was transferred to a surgical unit for surgical evaluation and underwent open-reduction internal fixation (ORIF) two days later.

**Assessment:** On assessment, this patient was considered a high fall risk at the time of the event. The patient was assisted to the bathroom by a nurse who left the patient on the toilet to go “respond to another patient yelling.” When the nurse turned to walk away, he heard the patient fall to the ground. The nurse failed to stay with the patient per hospital policy for patients who are high fall risk. This event has been classified as a serious safety event, noted as a preventable fall with harm.

**Recommendation:** This event meets Department of Public Health (DPH) Adverse Event (AE) reporting criteria. Root Cause Analysis (RCA) should be completed and reviewed by hospital committee.

**Answer the following questions:**

1. What are the steps to take in response to such an event?
2. Who should be involved in the response to such an event?

Instructions for Part II:

For Part II, you will break up into small groups to delve deeper into the use of a root cause analysis (RCA) to evaluate the preventable fall event. An RCA charter has been established, and a charter team has been formed to include the VP of Patient Services, the Clinical Program Manager, Subject Matter Expert (Falls Committee Chair), an RCA analyst, Medical Director, and Quality and Safety Director. Led by the VP of Patient Services, the charter team is tasked to explore and develop a plan for improvement related to the serious safety event.

Your small group will be given informational facts related to the case, as well as details about the different roles involved in the case. Each individual will take on an identified role, and be asked to roleplay using only the information given for the role. The group is tasked to meet together in order to work on detailing key events that took place in the safety event (both ideal events and actual events) and identify specific interventions for system improvement in order to prevent such events in the future.

**PART II**

**VP of Patient Services:** You are the Vice President of Patient Services, and are the sponsor the RCA. You hold responsibility for the execution and accountability related to the RCA process. You had been notified of this serious safety event within hours of the event occurring, and have tasked the RCA analyst to track and conduct interviews with appropriate stakeholders involved in the incident. In addition, you’ve worked with the appropriate individuals in patient relations in coordination with unit leadership to notify the family and patient about this event. The family and patient were understanding and did not decide to pursue legal action to the hospital.

You are particularly concerned about the application of the fall precaution policies of the hospital within this local inpatient unit, as this is not the first time that a serious safety event occurred on this inpatient unit. The hospital policy clearly states that for patients with high fall risk that a staff member should help with patient ambulation and toileting. In addition, given the fall event caused a hip injury which required surgery, the event needed to be reported as a serious safety event to the Department of Public Health. The hospital has been working at-large to reduce falls, and has chartered a falls committee as well as worked on numerous interventions to try to reduce falls. You plan to work with the unit leadership to identify key interventions that can be addressed to decrease hospital risk for future serious safety events as related to falls.

**RCA Analyst:**

You are the Root Cause Analysis Analyst. In your role, you’ve explored and conducted interviews with relevant stakeholders, including the nurse and other clinical staff members on the unit related to the falls event. When you discussed with the nurse, you found out the nurse was not normally rotating on the unit, and did not have as much familiarity with the unit culture. The nurse mentioned to you that he had acted out of concern for the other patient who was yelling out, and could not locate another nurse or member of the staff to help the other patient. He had inquired with the patient who was going to the bathroom whether the patient felt steady and could go, and the patient replied she felt “a little dizzy, but fine.” The nurse had not completed the fall risk score during that shift yet, and did not realize the patient was at high fall risk, since the information was not passed off to him on nursing handoff.

You had also interviewed the hospitalist attending, who mentioned that the patient was almost discharge-ready, and generally appeared stable after coming out of the ICU. The provider was planning to order physical therapy evaluation prior to discharge, in order to help determine disposition, but was awaiting a few more laboratory tests to return. The provider did not mention in the interview about medication changes even though a recent change in blood pressure medications could have potentially caused orthostatic hypotension, which was not ordered or checked.

**Medical Director:**

You are the Medical Director for the inpatient unit in which the fall event took place. You are a hospitalist by training and have oversight of the medical staff and resident training on the unit. You have historical context that the unit where the fall event took place has a history of fall events due to taking care of mostly elderly patients. The trend for falls has increased, despite staffing challenges to adequately staff both providers and nurses. This has been a challenge that you have experienced in your administrative role, and recognize could have contributed to this serious safety event.

In preparation for the RCA, you completed a chart audit on this patient. Upon review of the chart, you notice the patient has a history of being on Coumadin, and is anticoagulated for atrial fibrillation. You also realize the patient was recently started on a medication by one of the hospitalists that causes orthostatic hypotension but that there was not orthostatic vital signs ordered or completed for the patient. Following the event, the patient subjected provided history that she was dizzy prior to the bathroom, after getting up from bed, and lost balance on her way walking to the bathroom.

**Clinical Manager:**

You are the Clinical Manager, which means you have direct oversight of the nursing staff, unit clerks, and clinical technicians on the inpatient unit. You work collaboratively with the unit pharmacist and nursing leadership on patient quality and safety as well as unit operations. You review the fall event, and found that there were two less nursing staff and one less clinical technician on the shift, due to staff call-outs for sick leave. Although the nursing ratios were maintained, the staff involved in the fall event was responsible for more patients than typically designated. Although the nurse who had left the patient acted to respond to what he felt was an important issue of another patient calling out, the patient who was considered high risk was left alone. You had provided feedback to the nurse the next day, and also recognize the nurse typically works on another unit and may not have been as familiar with the unit where the fall event took place.

Upon review of the patient’s fall risk scoring scales, which was completed by several different nurses, you notice the patient was noted to score in the range of high-risk, then low-risk, and then high-risk, and wonder about the reliability of application of the fall risk scale in clinical practice and need for nursing re-education.

**Quality and Safety Director:**

You also have reviewed the patient chart as well as the hospital clinical dashboard for fall events, and found that the unit where the serious safety event had two other serious safety events earlier in the year, as well as numerous precursor and near-miss events related to falls. You found that on average, the unit experienced approximately 2-3 falls per week. Despite trying to identify Falls Champions and other invested individuals on falls prevention, the unit has had limited engagement with the hospital falls committee, and has not fully incorporated the best practices related to falls (i.e. reliably applying the fall risk scale, monitoring for orthostatic hypotension, referring patients for physical therapy and occupational therapy visits earlier in hospital stay). Your hope is that the unit leadership works together to identify and implement known, evidence-based strategies for falls prevention and are willing to work with the team on a quality improvement project related to falls reduction. However, you’ve been challenged by staffing shortages and lack of organized engagement related to falls from prior experience, and plan to utilize the RCA and this serious safety event as impetus to re-align and engage the unit leadership to act to address falls.

**Subject Matter Expert—Falls Committee Chair (Optional Role):**

You are the hospital Falls Committee Chair, and have been leading a group on quality improvement related to falls for the last 3 years. You are a geriatrician by training with background in quality and safety, and career interest in reducing falls in hospital care. Over that time period, you’ve worked with the team to develop metrics related to falls, including witnessed and unwitnessed falls, falls occurring due to poor handoffs, and timing of falls. You’ve also worked to develop a Falls Champions program, in which each clinical site has a designated champion in order to promote local quality/safety culture related to falls.

You help monitor the clinical dashboard related to falls for the hospital, and have noted increasing falls in the unit related to this serious safety event. At the RCA, you hope to provide content expertise as well as input on best practices related to falls prevention. Some of the common tips you typically offer on RCAs include improvement interventions to measure orthostatic vitals, monitoring medication changes, early referral to physical therapy/occupational therapy, nursing education and training, as well as provider and unit knowledge about falls prevention techniques (i.e. setting and responding to bed alarms, standardized tools for evaluating fall risk, consistent application of fall risk score, staffing adequacy to help patients who are at high fall risk).

**Answer the following questions:**

1. What are the causes that could have contributed to the serious safety events? (Consider domains related to individual factors, team factors, patient factors, environment, rules/policy, and organization)
2. What are areas for improvement and interventions to help prevent this event from happening again?
3. What are key lessons from this event?
