# Supplementary material for: Implementation of simulation-based health systems science modules for resident physicians
Source: BMC Med Educ. 2022 Jul 30;22:584. doi: 10.1186/s12909-022-03627-w (PMC9338604; doi:10.1186/s12909-022-03627-w)
Supplement: Supplementary file 2 — Additional file 2: Appendix 2. Post-survey template items [file 12909_2022_3627_MOESM2_ESM.docx]

**Appendix 2. Post-Survey Template Items**

- Do you feel you gained a better understanding of how [*insert module topic here*] following this session?

(5-point: Yes, absolutely; Yes, somewhat; Moderately; No, not really; No, not at all)

- - If moderate-yes: Please describe the specific ways your understanding of these concepts has changed.
  - If no: Please describe what we could do to improve our session to promote your understanding of these concepts.
- How useful were each of these elements in contributing towards your learning?

(5-point: Extremely useful; Not at all useful)

- Didactic PowerPoint
- Breakout Room Activity
- Large Group Debrief
- Other [write in]
- Did this session help you better understand [*insert module topic here*] in areas where you deliver care?
- Please describe why you selected your previous response. [write-in]
- What was the most helpful concept you learned or practiced during today's session? [write-in]
- What adjustments could we make to our process or content to improve your experience and its application to your work? [write-in]
- Which best describes your gender identity?

(Female; Male; Non-binary; Prefer to self-describe [write in]; Prefer not to say)

- Please select the race(s) with which you identify.

(American Indian or Alaskan Native; Asian; Black or African American; Hispanic or Latinx; Native Hawaiian or Other Pacific Islander; White; Prefer to self-describe [write in]; Prefer not to say)

(Optional) Please share anything else you believe would be helpful for us to know.
